# Diverse roles of lung macrophages in the immune response to influenza A virus

**DOI:** 10.3389/fmicb.2023.1260543

**Published:** 2023-09-13

**Authors:** Haoning Li, Aoxue Wang, Yuying Zhang, Fanhua Wei

**Affiliations:** ^1^College of Animal Science and Technology, Ningxia University, Yinchuan, China; ^2^School of Biological Science and Technology, University of Jinan, Jinan, China

**Keywords:** influenza virus, macrophage, antiviral, immune response, inflammation

## Abstract

Influenza viruses are one of the major causes of human respiratory infections and the newly emerging and re-emerging strains of influenza virus are the cause of seasonal epidemics and occasional pandemics, resulting in a huge threat to global public health systems. As one of the early immune cells can rapidly recognize and respond to influenza viruses in the respiratory, lung macrophages play an important role in controlling the severity of influenza disease by limiting viral replication, modulating the local inflammatory response, and initiating subsequent adaptive immune responses. However, influenza virus reproduction in macrophages is both strain- and macrophage type-dependent, and ineffective replication of some viral strains in mouse macrophages has been observed. This review discusses the function of lung macrophages in influenza virus infection in order to better understand the pathogenesis of the influenza virus.

## 1. Introduction

Influenza viruses include four major types A, B, C, and D, and belong to the family of *Orthomyxoviridae* family. Influenza A virus (IAV), influenza B virus (IBV), influenza C virus (ICV), and influenza D virus (IDV) can all infect mammals, with IAV, IBV, and ICV infecting humans. No human infections with IDV have been reported to date (Liu et al., 2020). Seasonal influenza viruses are responsible for most human respiratory infections, causing an estimated 290,000–650,000 deaths globally each year. IAVs are further divided into subtypes based on hemagglutinin (HA) and neuraminidase (NA) glycoproteins. There are 18 HA subtypes (H1–H18) and 11 NA subtypes (N1–N11) of IAV (Tong et al., 2013). Effective measures against IAV and IBV infection include the prevention of vaccine, or prophylactic or therapeutic treatment with antiviral drugs. However, the evolution of influenza viruses through antigenic drift and antigenic shift allows them to spread across different species and is responsible for the novel influenza viruses in pandemics (Taubenberger and Morens, 2008).

**Figure 1:**
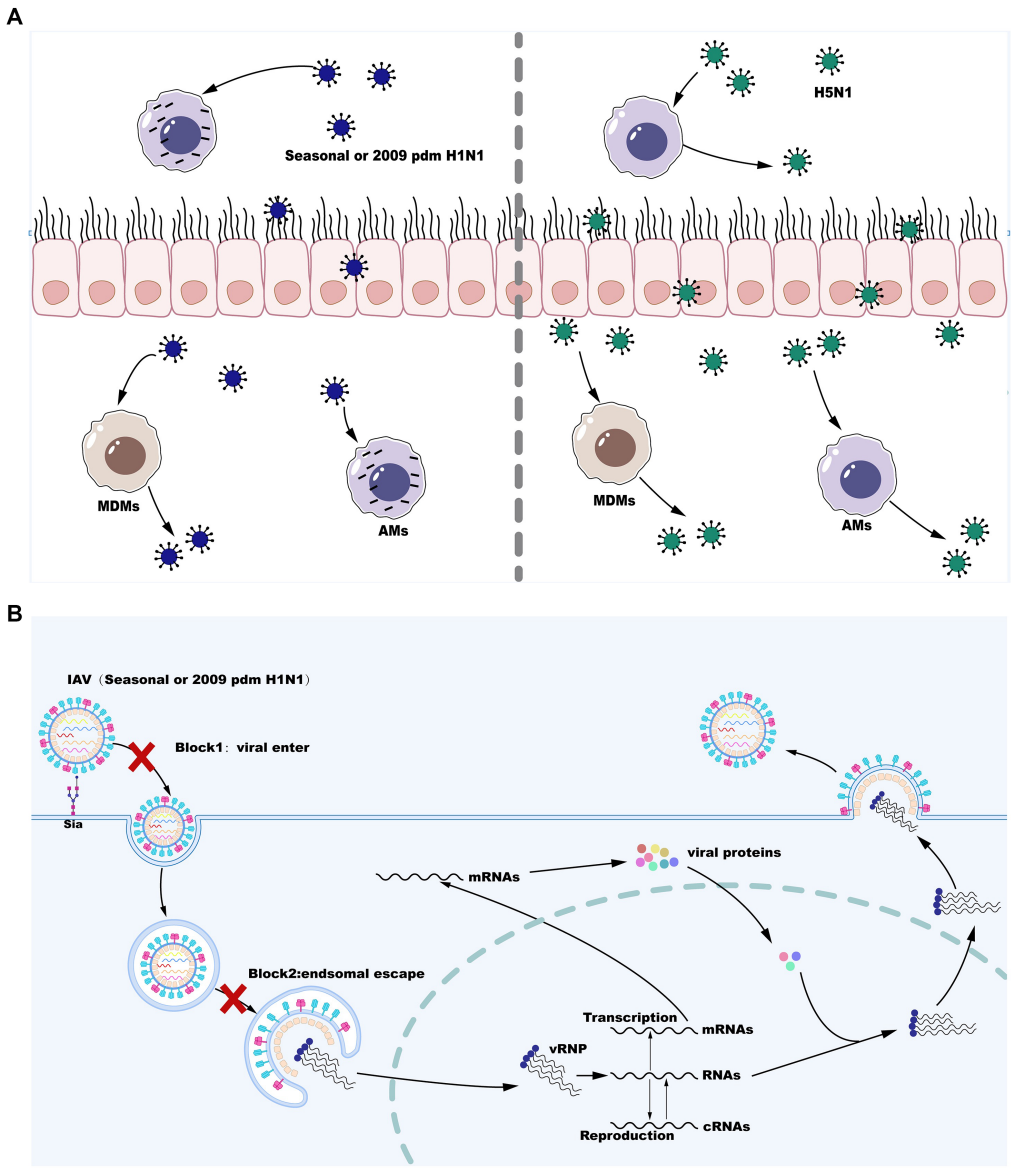
Influenza A virus (IAV) reproduction efficiency in MDMs and AMs. **(A)** Seasonal H1N1 or 2009 pdm H1N1 infection in MDMs is productively replicated, whereas seasonal H1N1 or 2009 pdm H1N1 infection in AMs is abortive. However, infection with some strains of H5N1 is productively replicated in both MDMs and AMs. **(B)** IAV replication blocks in AMs infection with seasonal H1N1 or 2009 pdm H1N1.

The innate immune system is the first line of defense against influenza virus infection consisting of physical barriers and innate cellular immune responses. During influenza virus infection, epithelial cells on the mucosa are infected and subsequently macrophages may be the cells that first responded to them immunologically and play a crucial role in virus resistance (Maines et al., 2008). Infection of respiratory epithelial cells with IAV is initiated by the binding of cell surface salivary acids to the viral HA proteins (Wiley and Skehel, 1987; Skehel and Wiley, 2000). Moreover, it is likely that viral entry into epithelial cells can be facilitated by the interaction with other cell surface receptors (Chu and Whittaker, 2004; Rapoport et al., 2006; Thompson et al., 2006; Oshansky et al., 2011). Alveolar macrophages (AMs) are also infected by seasonal IAV strains (H1N1 and H3N2) and replication of seasonal IAV in AMs is not productive (Rodgers and Mims, 1982; van Riel et al., 2011; Yu et al., 2011). However, certain viral strains that can replicate in a productive manner in macrophages are now well known and this has a significant impact on the antiviral functions of macrophages (Cline et al., 2017). Following attachment, virus is internalized by receptor-mediated endocytosis using clathrin- or caveolin-dependent or -independent pathways, and conformational changes of HA in the low pH of the endosomal compartment leads to the fusion of viral and endosomal membranes. The fusion of the viral envelope with the endosomal membrane triggers the release of vRNP to the cytoplasm before entry into the nucleus to initiate viral replication. Inefficient attachment and entry (Londrigan et al., 2015) and a block downstream of virus internalization but upstream of nuclear entry (Cline et al., 2013; Marvin et al., 2017) are two cellular blocks that regulate the productive replication of influenza virus in macrophages. However, the HPAI H5N1 viruses and the H1N1 WSN strains are able to overcome these cellular barriers and further replicate in a productive manner in both mouse and primary human macrophages (Cline et al., 2013; Marvin et al., 2017). Therefore, IAV replication in macrophages is both strain- and macrophage type-dependent. These are described below and are summarized in Figure 1.

## 2. Macrophages heterogeneity following IAV infection

### 2.1. Macrophages plasticity during IAV infection

Macrophages exist in the body as a heterogeneous population of cells. According to different physiological anatomical locations in the lungs, macrophages are classified as AMs and alveolar interstitial macrophages (IMs; Hume et al., 2020). In mice, AMs are characterized as major histocompatibility complex (MHC) class II^mod^CD11c^high^Siglec-F^high^ in contrast to MHC II^mod^CD11c^low^Siglec-F^neg^CD64^pos^ IMs (Misharin et al., 2013; Schneider et al., 2014b), whereas human AMs are MHC II^high^CD11c^high^CD14^low^ (Desch et al., 2016). Studies in granulocyte-macrophage colony-stimulating factor (GM-CSF)-deficient mice lacking functional Siglec-F^high^CD11c^high^ AMs demonstrate for the first time the requirement for AMs in lung homeostasis (Dranoff et al., 1994). Upon influenza virus infection, AMs are implicated in viral clearance since depletion of AMs leads to a higher viral load, an increase in mortality, and a reduction in the production of type I IFN (IFN-I; Tumpey et al., 2005; Schneider et al., 2014a). Importantly, the depletion of AMs in pigs also increases the mortality and loss of body weight following IAV infection (Kim et al., 2008). In addition, the frequency of IMs is found to be twice as low as that of AMs in mice under steady-state conditions (Bedoret et al., 2009), but IMs expand at a much faster rate when responding to external stimuli than AMs do (Landsman and Jung, 2007). Indeed, the H5N1 infection leads to the rapid depletion of AMs from both bronchoalveolar lavage (BAL) and the lungs and the concurrent recruitment of IMs to the lungs (Corry et al., 2022).

Alveolar macrophages can be further subdivided into resident alveolar macrophages (TR-AMs) and recruited monocyte-derived macrophages (MO-AMs), while IMs can be classified as TR-IMs and MO-IMs. Currently, studies of lung macrophages are focused on AMs, mainly due to technical problems in the extraction of IMs and their similar phenotype to monocytes (Liegeois et al., 2018). Macrophages from the yolk sac and fetal liver precursors enter various tissues during the formation of embryonic organs and differentiate into tissue-resident macrophages by transforming growth factor-β (TGF-β) and GM-CSF in adulthood (Guilliams et al., 2013; Yu et al., 2017; Zhao et al., 2018)^.^ In steady-state conditions, TR-AMs are cells with a relatively long life span, which are fully established before birth and are maintained by self-replication without the need for replenishment by blood monocytes (Yona et al., 2013). In contrast, lung MO-AMs have a limited ability of self-maintenance and are replenished by macrophages recruited from circulating inflammatory monocytes in the peripheral blood (Iwasaki, 2016). Hence, location and origin are the two principal determinants of macrophage characteristics in the lungs (Zhou and Moore, 2018). TR-AMs appear to limit their own plasticity due to their prolonged residence in the tissue to facilitate tissue homeostasis, whereas MO-AMs are more plastic to an inflammatory state (Guilliams and Svedberg, 2021). For example, CCR2-deficient mic with fewer circulating monocytes (Serbina and Pamer, 2006) are showing less susceptibility to influenza virus infection and lower morbidity and mortality (Lin et al., 2008). MO-AMs and TR-AMs have similar phenotypes in the early stages of influenza virus infection, but have different transcriptional and epigenetic profiles and show unique functions (MacLean et al., 2022). Within 1 month of influenza infection, MO-AMs are transcriptionally similar to monocytes and produce more interleukin-6 (IL-6) upon stimulation. After 2 months of influenza infection, recruited and resident AMs become similar in transcription and function, whereas MO-AMs lose plasticity and are not providing antimicrobial protection, playing a similar role to TR-AMs in balancing lipolysis to remove surfactant from the alveoli at steady-state conditions (Aegerter et al., 2020; Kulikauskaite and Wack, 2020).

### 2.2. Variable polarization of macrophages in influenza virus infection

Macrophages can be imprinted with different roles depending on the microenvironment. Upon influenza virus infection, macrophage polarization has occurred in the presence of environmental stimuli, in which activated macrophages can become M1 related to Th1 cytokine responses, or M2 relevant to Th2 cytokines. The phenotype of proinflammatory M1 is referred to as classically activated macrophages. M2 macrophages are referred to as alternatively activated macrophages and exert anti-inflammatory cell functions and boost tissue recovery (Stein et al., 1992). On the basis of the cytokines that induce M2 macrophages and their gene expression profiles, M2 macrophages can be further classified into the categories (M2a, b, c, and d; Mantovani et al., 2004). An overview of the types of polarization, secretory molecules, and major functions of M1 and M2 macrophages is given in Table 1.

**Table 1:** The polarization types, secreted molecules, and main functions of macrophages.

Phenotypes	Secreted molecules	Main functions
M1	IL-1β, IL-12, IL-23, IL-10, TNF, IL-6, iNOS, CCL2, CCL3, CCL4, CCL5, CCL9, and CCL10	Th1 responses; antimicrobial properties; and tumor resistance
M2a	Arg, IL-10, IL-1Ra, TGF-β, CCL17, and CCL22	Th2 responses; tissue remodeling; wound healing; and anti-inflammatory
M2b	IL-10, IL-12, TNF, IL-1β, IL-6, and CCL1	Th2 activation; immune regulation; tumor progression; and promoting infections
M2c	IL-10, TGF-β, and MerTK	Immune regulation; tissue remodeling; and phagocyte apoptotic cells
M2d	IL-10, VEGF	Angiogenesis; tumor progression

During the disease process, timely changes in the polarization status of M1/M2 macrophages are critical to the overall healing process. In early virus invasion, M1 macrophage dominance promotes an inflammatory response to clear pathogens, after which M2 type dominance can facilitate tissue healing (Alvarez et al., 2016). If M1 macrophages persistently survive predominantly, which results in immunopathological damage to the organism and impedes tissue healing (Alvarez et al., 2016). Influenza virus-induced GM-CSF decreases proinflammatory macrophages by re-directing macrophages to a more “M2-like” activation state from a “M1-like” type by altering the ratio of CXCL9 to CCL17 in BAL (Halstead et al., 2018). In A(H1N1), A(H3N2), and A(H9N2) virus infection, macrophages undergo M1 polarization at 4 hpi and M2b polarization at 8 hpi, which is modulated by the PI3K/Akt signaling (Zhao et al., 2014) and by the autophagy and exosome production (Xia et al., 2022). In contrast to H1N1 virus infection, a well-defined set of dysregulated genes and pathways that occurred in the M1 subtype is specific to H5N1 virus-infected macrophages, leading to an exacerbation of pathology of H5N1 infection (Zhang et al., 2018). However, the AMs polarization following influenza virus infection is multifactorial and complex. Influenza virus infection induces M1-polarized AMs in early stages and M2b-polarized AMs in the middle stage (Zhao et al., 2014). M1-polarized AMs have a lower endosomal pH and facilitate viral replication, whereas M2-polarized AMs have a higher endosomal pH but a lower lysosomal pH and restrict SARS-CoV-2 infection (Lv et al., 2021; Wang Z. et al., 2022). The M1 state of AMs toward the M2 state is associated with the increased severity and inflammatory responses to influenza virus infection (Lauzon-Joset et al., 2019). Therefore, characterizing the variations and ratios of M1 and M2 in the lungs with different doses and infection times contributes to regulating lung injury following influenza virus infection (Yao et al., 2022).

Macrophages differ in their differentiation status after influenza virus infection. M1 macrophages produce the proinflammatory TNF-α and iNOS, while M2 macrophages express IL-10 and TGF-β. M2 macrophages express the macrophage mannose receptor CD206, produce high levels of arginase-1 (Arg-1), and enhanced phagocytic capacity, which has a protective role in influenza virus infection (Gordon, 2003; Campbell et al., 2015). It has been demonstrated that M2 macrophages are more susceptible to apoptotic cell death than M1 macrophages following IAV infection (Campbell et al., 2015). Expression of the macrophage mannose receptor is upregulated in M2-polarized macrophages (Jablonski et al., 2015; Roszer, 2015), this may explain why M2 macrophages are more susceptible to IAV infection. Importantly, although IAV-infected M1 macrophages produce the highest expression of proinflammatory cytokines including TNF-α, IAV-infected M2 macrophages can override the anti-inflammatory cytokines profile of these cells and cause them to secret TNF-α and other M1-like cytokines. Interestingly, these findings are only observed during the infection of macrophages with HPAI H5N1 and WSN viruses (Cline et al., 2013; Marvin et al., 2017), which have been shown to replicate productively in macrophages, these findings may explain the hypercytokinemia and enhanced inflammatory response in severe H5N1 infection.

## 3. Regulating inflammation

### 3.1. The sentinel role of macrophages

**Figure 2:**
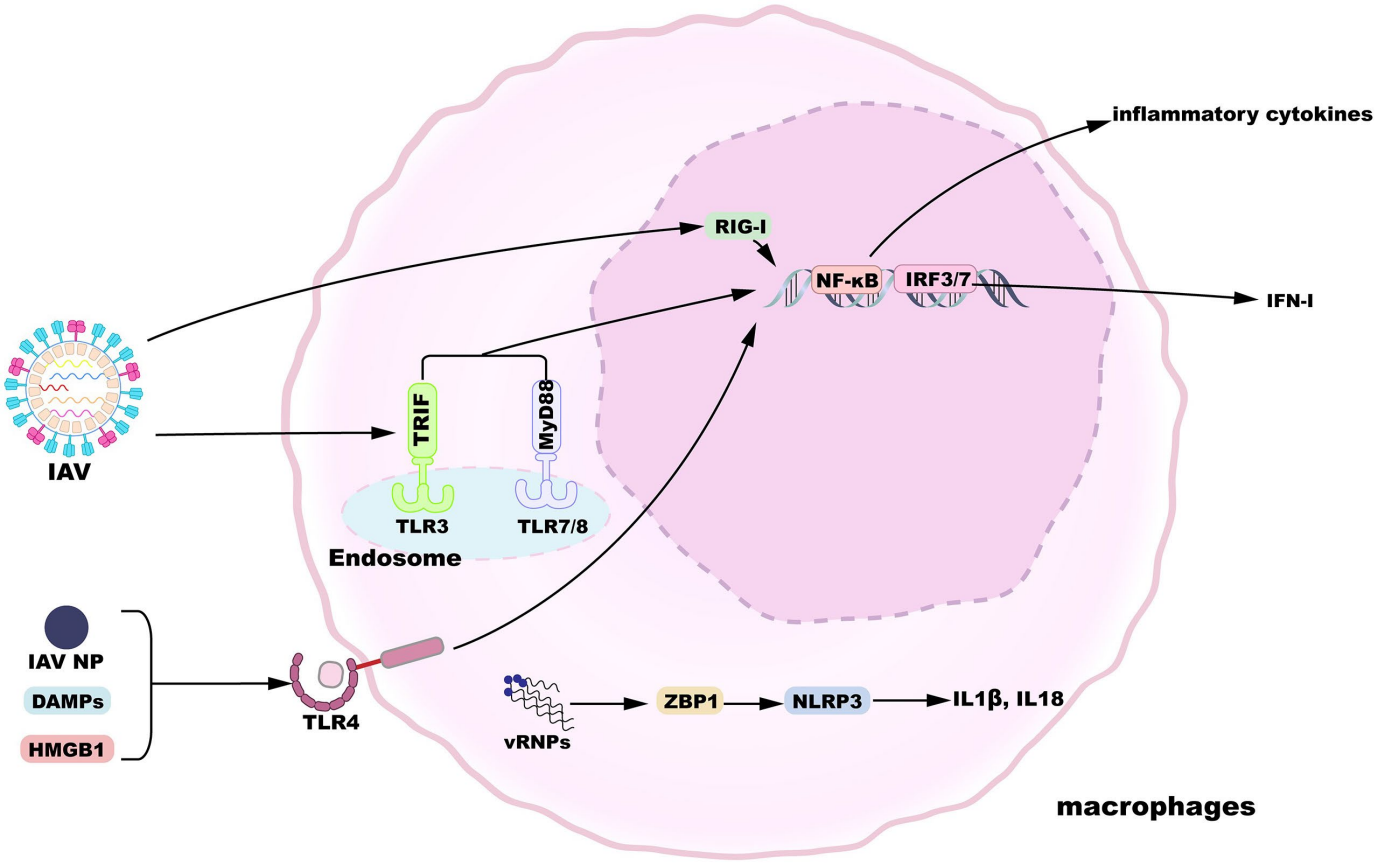
Macrophage sensing of influenza virus. TLR3, 7, and 8 can sense incoming virions. TLR4 is activated by HMGB1 or IAV nucleoprotein. TLR engagement results in the activation of proinflammatory or antiviral gene expression downstream of NF-κB or IRF3/7 signaling pathways. Additionally, replication products of IAV are recognized by nuclear RIG-I or ZBP1, inducing gene expression.

Alveolar macrophages display a state of relative quiescence, produce cytokines at low levels, and inhibit innate and adaptive immunity induction in the absence of infection (Hussell and Bell, 2014). After the influenza virus invades the epithelial barrier, AMs are the innate immune cells that can respond in the early stages of infection to initiate and evoke innate immunity. In addition to their vital phagocytic function, AMs also encode a variety of pattern-recognition receptors (PRRs) essential for the sensing of pathogens and tissue damage (Janeway and Medzhitov, 2002). Toll-like receptor (TLR) 3 is expressed by macrophages and recognizes viral RNA structures in phagocytosed IAV-infected cells (Schulz et al., 2005). TLR3 activation induces the upregulated expression of nuclear factor κB (NF-κB)-regulatory proinflammatory cytokines as well as the expression of IFN-I and IFN-stimulated genes (ISGs) regulated by interferon regulatory factor 3 (IRF3). When IFN-I binds to the IFNα/β receptor (IFNAR), a heterodimer consisting of two subunits, IFNAR1 and IFNAR2, it activates the Janus kinase/signal transducers and activators of transcription (JAK/STAT) pathway to induce the expression of ISGs, thereby inhibiting viral replication (Makris et al., 2017). Meanwhile, macrophages-expressed TLR7 and TLR8 sense IAV ssRNA liberated from engulfed cells (Diebold et al., 2004; Lund et al., 2004; Iwasaki and Pillai, 2014), which engage the viral RNA signal in a manner dependent on myeloid differentiation primary response gene 88 (MyD88) and then activate transcription factors NF-κB and IRF7. Following influenza virus infection, IAV proteins and nucleic acids produced by apoptotic cells are released into the extracellular space. For instance, IAV infection activates TLR4 pathway in lung macrophages by recognizing IAV nucleoprotein (NP), though whether NP triggers IFN-β production via TLR4 is not measured (Kim et al., 2022). Accumulating results suggest that and several host-derived damage-associated molecular patterns (DAMPs) can activate TLR4 pathway and trigger proinflammatory cytokines release, such as high-mobility group box 1 protein (HMGB1) and oxidized phospholipids (Imai et al., 2008; Shirey et al., 2016; Bertheloot and Latz, 2017). Moreover, RNA intermediates of IAV replication can be sensed by retinoic-acid inducible gene I (RIG-I) in the nucleus, resulting in the expression of IFN-I and proinflammatory cytokines (Liu et al., 2018). Viral ribonucleoproteins (vRNPs) are potentially recognized by Z-DNA-binding protein 1 (ZBP1), which triggers the activation of NLRP3 inflammasome to release IL-1β and IL-18 (Kuriakose et al., 2016). An overview of macrophage sensing implicated in IAV infection is provided in Figure 2.

It has been demonstrated that primary human macrophages can be infected by the avian H5N1 and seasonal H1N1 influenza viruses (Lee et al., 2009) and murine BMDMs can also be infected by the H1N1 and H5N1 viruses efficiently (Chan et al., 2012). Upon influenza virus infection, macrophages produced IFN-I and other cytokines including type III IFNs, IL-12, IL-1β, tumor necrosis factor-alpha (TNF-α), IL-6, and several chemokines are induced through different pathways. Responsiveness of macrophages and induction of IFN-I play a crucial role in protecting the lower respiratory tract, limiting viral spread, and effective immune protection against influenza virus infection. For instance, following influenza virus infection, AMs-produced IFN-I plays a role of immune protection through the promotion of hematopoietic cell proliferation and differentiation (Essers et al., 2009; Sato et al., 2009) and the upregulation of chemokine (C-C motif) ligand 2 (CCL2), which is necessary for CCR2-dependent Ly6C^hi^ monocyte egress from the bone marrow. However, IFNα/β IFNα/β may be exacerbated during virus infection by hindering viral control (Guarda et al., 2011; Teijaro et al., 2013) or by causing inflammation and tissue damage that exacerbate the disease (Hogner et al., 2013; Davidson et al., 2014). Moreover, IFN-γ is a major cytokine associated with M1 activation and is also involved in the M1/M2 paradigm together with LPS. The M1/M2 polarization following influenza virus infection will be discussed in the other section. In addition, IFN-λ is produced earlier and more frequently than IFN-I in influenza infection, which establishes the cellular state of viral resistance and induces a similar ISGs signature. Because of the abundant expression of IFN-λ in the initial stage of infection, IFN-λ signaling in macrophages likely plays a crucial role in combating influenza infection (Mallampalli et al., 2021). During influenza virus infection, IFN-λ is produced mainly by alveolar type II epithelial cells (ATIIs; Wang et al., 2009) and limits virus infection in respiratory and gastrointestinal epithelial cells since the expression of functional interferon λ receptor (IFNLR) complexes in the lungs and intestine is confined to epithelial cells (Mordstein et al., 2010). However, expression of IFNLR1 in macrophages is species-specific since macrophage colony-stimulating factor (M-CSF) or GM-CSF stimulation fails to induce ISGs after IFNL2 or IFNL3 stimulation in mice (Mallampalli et al., 2021). IFN-λ production reduces viral release and suppresses influenza virus-induced secretion of inflammatory cytokines including chemokines and IFN-β (Wang et al., 2009), facilitates the protective immune response of IAV-specific CD8+ T cells (Hemann et al., 2019), and promotes the proliferation and maturation of natural killer cells (Wang et al., 2017), with relatively fewer inflammatory side effects than IFN-α (Davidson et al., 2016).

### 3.2. The role of macrophages in the induction of excessive inflammatory response

Following influenza virus infection, the severity of the disease depends on the virulence of the influenza virus and host factors (Liu et al., 2016). In the 1918 H1N1 or the H5N1 viruses infection, cytokines and chemokines are excessively induced, causing a hypercytokinemia or “cytokine storm” that results in histopathological changes, systemic sepsis, and multi-organ dysfunction (Tisoncik et al., 2012; Liu et al., 2016; Wei et al., 2022). Recruitment of monocytes and expression of proinflammatory cytokines are upregulated in young mice during IAV infection, with increased secretion of IFN-I, monocyte chemotactic protein 1 (MCP-1), excessive recruitment of inflammatory monocytes, and persistent activation of NLR family pyrin domain containing 3 (NLRP3), resulting in increased mortality in young mice (Coates et al., 2018). Further studies have found that mice lacking NO synthase 2 (NOS2) or TNF have a decreased mortality during influenza virus infection, and that recruited macrophages are the major producers of inducible nitric oxide synthase (iNOS) production and directly responsible for NOS2 and TNF production, maybe the main cell population responsible for immunopathology (Karupiah et al., 1998; Jayasekera et al., 2006; Lin et al., 2008).

Macrophages can cause pathological immunity following influenza virus infection but also secrete cytokines to counteract the over-reactive inflammatory response. IL-10 is a robust anti-inflammatory factor mainly derived from macrophages, which inhibits the overproduction of inflammatory cytokines during infection or tissue injury through negative feedback regulation (Iyer et al., 2010). Inflammatory cytokines comprising IL-1β and TNF are produced in large quantities early in influenza virus infection, and homeostasis-related cytokines including IL-10 emerge later to inhibit the innate immune inflammatory response (Tisoncik et al., 2012; Wang et al., 2012). In addition to IL-10, macrophage-sourced peroxisome proliferator-activated receptor γ (PPAR-γ) suppresses overstated antiviral and inflammatory reactions caused by influenza virus infection (Huang et al., 2019). Level of PPAR-γ is downregulated in macrophages through IFN-dependent signaling during influenza virus infection, whereas expression of genes related to healing injured tissue in late disease such as endothelial and epithelial cell growth factors, is blocked in mice lacking PPAR-γ, suggesting that macrophage-derived PPAR-γ promotes tissue repair (Huang et al., 2019). Transcription factors also play a key role in regulating the inflammatory and reparative effects of AMs after infection, such as β-catenin and HIF-1α, and deletion of these transcription factors in AMs results in accelerated rates of inflammation and lung repair (Zhu et al., 2021). In addition to AMs, IMs also exhibit another activation phenotype and play an immunomodulatory role in controlling excessive lung inflammation following influenza infection (Ural et al., 2020).

## 4. IAV infection and macrophage phagocytosis

As professional phagocytes, macrophages perform an instrumental role in clearing infectious organisms through the internalization and degradation of pathogens and by phagocytosis of apoptotic cells. Phagocytosis can be mediated through the phagocytic receptors on the surface of macrophages including mannose receptors, scavenger receptors (SRs), complement receptors, macrophage receptors with collagenous structure (MARCO), CD36, and Fc receptors. Phagocytosis by macrophages of apoptotic IAV-infected cells inhibits further transmission of the virus and facilitates disease control (Watanabe et al., 2005). When the IAV-infected epithelial cells undergo apoptosis, they can be effectively phagocytosed by macrophages at an early stage, which leads to the inhibition of virus growth (Fujimoto et al., 2000). Similarly, IAV-infected mice-derived BAL macrophages incorporate phagocytosed apoptotic cells and IAV-infected mice-derived AMs exhibit enhanced engulf capacity than uninfected mice-derived AMs (Watanabe et al., 2005; Hashimoto et al., 2007). The influenza virus-infected mice are treated with phagocytosis inhibitors, resulting in decreased levels of phagocytosis with BAL cells and increased lethality and lung inflammation in those mice (Watanabe et al., 2005). Further studies have demonstrated that phosphatidylserine (PS) and carbohydrate molecules on the macrophage surface mediate the process of phagocytosis of influenza virus-infected cells, and these molecules are modified by influenza NA expressed in virus-infected cells (Shiratsuchi et al., 2000; Watanabe et al., 2002). During virus-infected cell apoptosis, PS located in the inner layer of the plasmatic membrane is exposed on the cell surface and acts as a potent prophagocytic signal to attract phagocytes (Shiratsuchi and Nakanishi, 2006; Birkle and Brown, 2021). In steady-state conditions, “Do not eat me” signal is activated and prevents cells from being phagocytosed, while virus infection causes a profound reduction of surface silicic acid residues on live influenza virus-infected cells (Nita-Lazar et al., 2015), which promotes phagocytosis of the cells (Meesmann et al., 2010). During influenza virus infection, virus-infected alveolar type II cells and epithelial cells can release the find-me signals to direct macrophages toward their positions and facilitate their phagocytoses, such as C-X-C Motif chemokine ligand 10 (CXCL10) and other chemokines including CXCL8/12 (Birkle and Brown, 2021). Furthermore, the complement system and PPRs also play an important role in rapidly mobilizing macrophages to the destination location shortly after the infection with the influenza virus and promote phagocyte recognition (Birkle and Brown, 2021). Therefore, apoptosis of cells infected with the influenza virus causes phagocytosis of such cells, and direct clearance of the virus function as a mechanism for the antiviral immune response.

However, excessive clearance is detrimental to the inflammatory response. Further studies have demonstrated that MARCO plays a deleterious role for in early influenza viral immune responses (Ghosh et al., 2011). The enhanced proinflammatory gene induction in macrophages is observed in MARCO-deficient macrophages, and MARCO-deficient mice infected with IAV show improved survival and earlier relief from weight loss and morbidity symptoms (Ghosh et al., 2011). This is mainly due to the removal of proinflammatory oxidized lipoproteins from cellular debris by MARCO, which inhibits the early inflammatory response (Ghosh et al., 2011). Oxidized phospholipids are recognition signals released by apoptotic cells that promote phagocytosis of apoptotic cells, while inducing several proinflammatory genes such as MCP1 and IL-8 facilitate the resolution of acute inflammation (Kadl et al., 2004). Furthermore, it has been revealed that after sensing pathogens, macrophages can crawl through the pores of Kohn between alveoli and engulf pathogens with high phagocytic efficiency, whereas influenza virus infection impairs macrophages crawling through IFN-γ signaling, causing inappropriate inflammation and injury by excessive induction of neutrophil and increasing secondary bacterial infection (Neupane et al., 2020). Additionally, AMs have higher levels of phagocytic activity and faster phagocytic processes than IMs, while IMs located in interstitial locations with lower phagocytic capacity can engulf pathogens that escape from AMs, thus reducing the spread of pathogens within the organism (Fathi et al., 2001).

The phagocytic capacity of macrophages is also affected following influenza virus infection, and macrophage phagocytic activity against apoptotic cells is increased *in vitro* when macrophages are incubated with the supernatant from IAV-infected epithelial cells (Hashimoto et al., 2007). And cells infected with the influenza virus undergoing apoptosis can release heat-labile substances that stimulate the phagocytic activity of macrophages (Hashimoto et al., 2007). Environmental pollutants, cigarette smoke, and alcohol also can inhibit the phagocytosis of macrophages (Karavitis and Kovacs, 2011). Moreover, older adults have comparatively lower macrophage numbers and reproductive capacity, and exhibit higher mortality after influenza virus infection due to selective downregulation of the clearance receptor CD204 (Wong et al., 2017). The AMs of offspring of pregnant mice infected with the influenza virus have a reduced ability to clear influenza B virus and MRSA and are more susceptible to influenza virus infection (Jacobsen et al., 2021).

## 5. IAV-induced secondary bacterial pneumonia involves lung macrophages

Secondary bacterial pneumonia is another dominant cause of fatalities caused by IAV infection (Smith and McCullers, 2014) and is the main cause of human mortality for the 1918 Spanish flu (Morens et al., 2008). Many studies have shown that the progression of secondary bacterial infections is regulated by IAV proteins. For example, non-structural protein 1 (NS1) affects the regulation of the interferon response and its motif acts directly in influenza virus and *S. pneumoniae* co-infections (Shepardson et al., 2019). NA also facilitates access to receptors and nutrients for *S. pneumoniae* and thereby promotes the development of bacterial infection (Feng et al., 2013; Siegel et al., 2014), whereas matrix 1 protein (M1; Halder et al., 2011), NP (Tripathi et al., 2013), M2 (Ichinohe et al., 2010), and HA (Klonoski et al., 2018) indirectly help in secondary bacterial infection development. In addition to functional defects, influenza virus infection decreases the number of AMs, resulting in increased susceptibility to bacterial superinfections (Ghoneim et al., 2013).

Multiple pathways of secondary bacterial pneumonia caused by IAV infection in relation to lung macrophages have been reported. As a preliminary point, AMs susceptibility to IAV-induced apoptosis promotes secondary bacterial infection by reducing anti-microbial lung macrophages (Ghoneim et al., 2013). This is also supported by the findings that the risk of secondary pneumococcal pneumonia decreased on the 14th day after infection with IAV, coinciding with a recovery in the number of AMs (Ghoneim et al., 2013). Moreover, inappropriate expression of cytokines can increase the risk of secondary bacterial infections by modulating macrophages. For example, IL-27 regulates the enhancement of *S. aureus* pneumonia susceptibility after influenza virus infection through the induction of IL-10 and the inhibition of IL-17 (Robinson et al., 2015). IFN-I production induced by IAV infection also enhances the susceptibility to secondary bacterial pneumonia because influenza-infected IFNAR−/− mice show increased survival and an increased capacity to clear secondary *S. pneumoniae* infection (Shahangian et al., 2009). In another study, IAV-induced IFN-γ inhibits the expression of MARCO by macrophages, thereby further inhibiting the uptake and killing *S. pneumoniae* during superinfections (Sun and Metzger, 2008). Additionally, influenza virus infection also chronically inhibits the ability of AMs to respond to TLR ligands. TLR9 expression on the surface of macrophages is upregulated after IAV infection (Martinez-Colon et al., 2019). It has been reported that TLR9-deficient mice show an increased expression of scavenger receptor A and iNOS on macrophages and an increased phagocytosis and killing of bacteria after influenza virus infection (Martinez-Colon et al., 2019). Anyway, these studies are mainly based on the findings that did not consider the lung microbiota, so it is imperative to include the role of the respiratory microbiome in influenza and secondary bacterial superinfections.

## 6. Involved in adaptive immune responses

Adaptive response, including CD4+ Th cells, CD8+ cytotoxic T lymphocytes (CTLs), B cells, and antigen-specific antibodies. Antigen-presenting cells (APCs) take up foreign substances and further degraded and presented them to T cells via MHC I or II molecules, or to T cells via other APCs. Endogenously processed viral peptides are presented by MHC I molecules on virus-infected cells surface and recognized by naive CD8+ T cells, which induces naive CD8+ T cells to differentiate into cytotoxic T cells that can recognize and kill virus-infected cells, whereas naive CD4+ T cells recognize exogenously processed peptides presented by MHC II molecules and differentiate through different pathways to generate effector subpopulations with different immune functions such as Th1 cells, Th2 cells, T follicular helper (Tfh), Th17 cells, and regulatory T cells (Tregs), which regulate the activation of target cells (Jin et al., 2012; Roche and Furuta, 2015). Th1 cells in the respiratory tract are activated following influenza virus infection, resulting in the production of TNF-α, IL-2, and IFN-γ (Zhu et al., 2010). Although T cell-mediated immune responses have a limited role in preventing initial viral replication, IFN-γ-producing Th1 cells are necessary for CD8+ T-cell activation and clearance of influenza virus and in preparation for recurrent infection. After influenza virus infection, Th2 cell-mediated immune responses aggravate lung tissue injury and postpone viral elimination (Graham et al., 1994). Highly specific and memorized humoral responses require the involvement of Tfh cells, whereas Th17 cells are involved in viral pathogenicity because influenza antigens-induced Th17 cells increase lung inflammation and morbidity after influenza virus challenging (Maroof et al., 2014; Miyauchi, 2017). However, other studies have suggested that the adoptive transfer of Th17 cells protects naive mice from a deadly flu challenge and defense against bacterial infections following influenza virus infection (McKinstry et al., 2009; Kudva et al., 2011). Furthermore, IAV stimulates the migration of Tregs into the lungs, this leads to a reduction in the cell number of Th17 cells and infiltrated neutrophils as well as diminished lung inflammation (Egarnes and Gosselin, 2018). Collectively, the viral sensing system detects influenza virus infection and releases chemokines to facilitate the migration of naive T cells, induce cytokines signaling, and convert APCs to effector T cells.

Antigen-presenting cells are important mediators that bridge the innate immune responses and adaptive immune system. The major APCs in activated naive T cells are DCs, which are the main initiating APCs and are induced to move toward local lymphatic tissues to present antigens and activate naive T cells under infection and inflammation conditions (Itano and Jenkins, 2003). Unlike DCs, tissue-resident macrophages are usually non-migratory and virus invasion does not cause them to migrate to lymphoid tissue, and therefore AMs have a limited role in antigen presentation and mostly maintain homeostasis of the lungs (Itano and Jenkins, 2003; Hou et al., 2021). Macrophages may be more important for the local amplification of T-cell responses already initiated by DCs. Macrophages rely on phagocytosis for antigen uptake, phagocytose and degrade them into peptides for presentation, while intracellular signaling is triggered by recognition of influenza virus by TLRs (Roche and Furuta, 2015; Koutsakos et al., 2019). Importantly, IMs are found to be more competent than AMs in driving T cell responses (Zaynagetdinov et al., 2013).

Efficacious humoral responses in the period of influenza virus infection encompass the induction of virus-specific neutralizing antibodies against viral HA and NA in an attempt to block infection, which is predominantly strain-specific (Waffarn and Baumgarth, 2011). It has been demonstrated that macrophages also promote humoral immunity. Recent studies have revealed that influenza virus rechallenge induces resident memory B (RMB) cell mobility and fast relocation to infected sites, subsequently differentiates into plasma cells and therefore leads to increased local antibody concentrations, this process is mediated by AMs, partly due to the induction of CXCL9 and CXCL10 expression (MacLean et al., 2022). These findings suggest that it will be useful to integrate strategies for inducing the concentration of cross-reactive antibodies at or near the location of viral entry (Iwasaki, 2016). Antigenic exposure at mucosal sites directly induces innate immune memory in tissue-resident macrophages populations, which are a new vaccine target for the development a novel type of adjuvants and of respiratory mucosal vaccine against influenza virus (Xing et al., 2020; Wang X. et al., 2022).

## 7. Conclusions and future perspective

The uncontrolled spread of influenza viruses places a burden on the public health of society and creates a serious threat to human life and health. The most seasonal and low pathogenic strains of the influenza virus cause an abortive infection in macrophages and therefore contribute to effective host defense, whereas some highly pathogenic strains of IAV infect macrophages productively. Given the diversity and plasticity of macrophages, elucidating the complicated interactions between macrophage phenotype and influenza virus infection will be helpful for deciphering the mechanisms implicating severe influenza disease. The development of single cell omics has led to new information regarding lung macrophages subtypes (Aegerter et al., 2022). Moreover, the advent of newer technologies of lineage tracing have improved our understanding of the plasticity of macrophages during and after infection, particularly in relation to AM subsets that originate from yolk sac macrophages versus MDMs. In the future, we can identify host restriction factors that determine abortive versus productive infection in macrophage types and distinguish different macrophages, and define biological agents that regulate the migration and differentiation of lung macrophages following influenza virus infection, these studies are also likely to provide new solutions for treatment and therapy options.

## Author contributions

FW: Conceptualization, Project administration, Supervision, Writing – original draft, Writing – review & editing. HL: Writing – original draft, Writing – review & editing. YZ: Conceptualization, Supervision, Writing – original draft, Writing – review & editing. AW: Writing –original draft, Writing – review & editing.

## Funding

The author(s) declare financial support was received for the research, authorship, and/or publication of this article. This work was supported by the Ningxia Natural Science Foundation of China (2021AAC05006) and the National Natural Science Foundation of China (NSFC; 31972669 and 81960297).

## Conflict of interest

The authors declare that the research was conducted in the absence of any commercial or financial relationships that could be construed as a potential conflict of interest.

## Publisher’s note

All claims expressed in this article are solely those of the authors and do not necessarily represent those of their affiliated organizations, or those of the publisher, the editors and the reviewers. Any product that may be evaluated in this article, or claim that may be made by its manufacturer, is not guaranteed or endorsed by the publisher.

## References

[ref1] Aegerter H. Kulikauskaite J. Crotta S. Patel H. Kelly G. Hessel E. M.. (2020). Influenza-induced monocyte-derived alveolar macrophages confer prolonged antibacterial protection. Nat. Immunol. 21, 145–157. doi: 10.1038/s41590-019-0568-x, PMID: 31932810 PMC6983324

[ref2] Aegerter H. Lambrecht B. N. Jakubzick C. V. (2022). Biology of lung macrophages in health and disease. Immunity 55, 1564–1580. doi: 10.1016/j.immuni.2022.08.010, PMID: 36103853 PMC9533769

[ref3] Alvarez M. M. Liu J. C. Trujillo-de Santiago G. Cha B. H. Vishwakarma A. Ghaemmaghami A. M.. (2016). Delivery strategies to control inflammatory response: modulating M1-M2 polarization in tissue engineering applications. J. Control. Release 240, 349–363. doi: 10.1016/j.jconrel.2016.01.026, PMID: 26778695 PMC4945478

[ref4] Bedoret D. Wallemacq H. Marichal T. Desmet C. Quesada Calvo F. Henry E.. (2009). Lung interstitial macrophages alter dendritic cell functions to prevent airway allergy in mice. J. Clin. Invest. 119, 3723–3738. doi: 10.1172/JCI39717, PMID: 19907079 PMC2786798

[ref5] Bertheloot D. Latz E. (2017). HMGB1, IL-1alpha, IL-33 and S100 proteins: dual-function alarmins. Cell. Mol. Immunol. 14, 43–64. doi: 10.1038/cmi.2016.34, PMID: 27569562 PMC5214941

[ref6] Birkle T. Brown G. C. (2021). I'm infected, eat me! Innate immunity mediated by live, infected cells Signaling to be phagocytosed. Infect. Immun. 89:e00476-20. doi: 10.1128/IAI.00476-20, PMID: 33558325 PMC8091084

[ref7] Campbell G. M. Nicol M. Q. Dransfield I. Shaw D. J. Nash A. A. Dutia B. M. (2015). Susceptibility of bone marrow-derived macrophages to influenza virus infection is dependent on macrophage phenotype. J. Gen. Virol. 96, 2951–2960. doi: 10.1099/jgv.0.000240, PMID: 26297234 PMC4635478

[ref8] Chan R. W. Leung C. Y. Nicholls J. M. Peiris J. S. Chan M. C. (2012). Proinflammatory cytokine response and viral replication in mouse bone marrow derived macrophages infected with influenza H1N1 and H5N1 viruses. PLoS One 7:e51057. doi: 10.1371/journal.pone.0051057, PMID: 23226456 PMC3511392

[ref9] Chu V. C. Whittaker G. R. (2004). Influenza virus entry and infection require host cell N-linked glycoprotein. Proc. Natl. Acad. Sci. U. S. A. 101, 18153–18158. doi: 10.1073/pnas.0405172102, PMID: 15601777 PMC535801

[ref10] Cline T. D. Beck D. Bianchini E. (2017). Influenza virus replication in macrophages: balancing protection and pathogenesis. J. Gen. Virol. 98, 2401–2412. doi: 10.1099/jgv.0.000922, PMID: 28884667 PMC5725990

[ref11] Cline T. D. Karlsson E. A. Seufzer B. J. Schultz-Cherry S. (2013). The hemagglutinin protein of highly pathogenic H5N1 influenza viruses overcomes an early block in the replication cycle to promote productive replication in macrophages. J. Virol. 87, 1411–1419. doi: 10.1128/JVI.02682-12, PMID: 23152519 PMC3554171

[ref12] Coates B. M. Staricha K. L. Koch C. M. Cheng Y. Shumaker D. K. Budinger G. R. S.. (2018). Inflammatory monocytes drive influenza a virus-mediated lung injury in juvenile mice. J. Immunol. 200, 2391–2404. doi: 10.4049/jimmunol.1701543, PMID: 29445006 PMC5860989

[ref13] Corry J. Kettenburg G. Upadhyay A. A. Wallace M. Marti M. M. Wonderlich E. R.. (2022). Infiltration of inflammatory macrophages and neutrophils and widespread pyroptosis in lung drive influenza lethality in nonhuman primates. PLoS Pathog. 18:e1010395. doi: 10.1371/journal.ppat.1010395, PMID: 35271686 PMC8939778

[ref14] Davidson S. Crotta S. McCabe T. M. Wack A. (2014). Pathogenic potential of interferon alphabeta in acute influenza infection. Nat. Commun. 5:3864. doi: 10.1038/ncomms4864, PMID: 24844667 PMC4033792

[ref15] Davidson S. McCabe T. M. Crotta S. Gad H. H. Hessel E. M. Beinke S.. (2016). IFNlambda is a potent anti-influenza therapeutic without the inflammatory side effects of IFNalpha treatment. EMBO Mol. Med. 8, 1099–1112. doi: 10.15252/emmm.201606413, PMID: 27520969 PMC5009813

[ref16] Desch A. N. Gibbings S. L. Goyal R. Kolde R. Bednarek J. Bruno T.. (2016). Flow cytometric analysis of mononuclear phagocytes in nondiseased human lung and lung-draining lymph nodes. Am. J. Respir. Crit. Care Med. 193, 614–626. doi: 10.1164/rccm.201507-1376OC, PMID: 26551758 PMC4824940

[ref17] Diebold S. S. Kaisho T. Hemmi H. Akira S. Reis e Sousa C. (2004). Innate antiviral responses by means of TLR7-mediated recognition of single-stranded RNA. Science 303, 1529–1531. doi: 10.1126/science.1093616, PMID: 14976261

[ref18] Dranoff G. Crawford A. D. Sadelain M. Ream B. Rashid A. Bronson R. T.. (1994). Involvement of granulocyte-macrophage colony-stimulating factor in pulmonary homeostasis. Science 264, 713–716. doi: 10.1126/science.8171324, PMID: 8171324

[ref19] Egarnes B. Gosselin J. (2018). Contribution of regulatory T cells in nucleotide-binding oligomerization domain 2 response to influenza virus infection. Front. Immunol. 9:132. doi: 10.3389/fimmu.2018.00132, PMID: 29445379 PMC5797787

[ref20] Essers M. A. Offner S. Blanco-Bose W. E. Waibler Z. Kalinke U. Duchosal M. A.. (2009). IFNalpha activates dormant haematopoietic stem cells in vivo. Nature 458, 904–908. doi: 10.1038/nature07815, PMID: 19212321

[ref21] Fathi M. Johansson A. Lundborg M. Orre L. Skold C. M. Camner P. (2001). Functional and morphological differences between human alveolar and interstitial macrophages. Exp. Mol. Pathol. 70, 77–82. doi: 10.1006/exmp.2000.2344, PMID: 11263950

[ref22] Feng C. Zhang L. Nguyen C. Vogel S. N. Goldblum S. E. Blackwelder W. C.. (2013). Neuraminidase reprograms lung tissue and potentiates lipopolysaccharide-induced acute lung injury in mice. J. Immunol. 191, 4828–4837. doi: 10.4049/jimmunol.1202673, PMID: 24068662 PMC3839962

[ref23] Fujimoto I. Pan J. Takizawa T. Nakanishi Y. (2000). Virus clearance through apoptosis-dependent phagocytosis of influenza a virus-infected cells by macrophages. J. Virol. 74, 3399–3403. doi: 10.1128/JVI.74.7.3399-3403.2000, PMID: 10708457 PMC111841

[ref24] Ghoneim H. E. Thomas P. G. McCullers J. A. (2013). Depletion of alveolar macrophages during influenza infection facilitates bacterial superinfections. J. Immunol. 191, 1250–1259. doi: 10.4049/jimmunol.1300014, PMID: 23804714 PMC4907362

[ref25] Ghosh S. Gregory D. Smith A. Kobzik L. (2011). MARCO regulates early inflammatory responses against influenza: a useful macrophage function with adverse outcome. Am. J. Respir. Cell Mol. Biol. 45, 1036–1044. doi: 10.1165/rcmb.2010-0349OC, PMID: 21562316 PMC3262690

[ref26] Gordon S. (2003). Alternative activation of macrophages. Nat. Rev. Immunol. 3, 23–35. doi: 10.1038/nri978, PMID: 12511873

[ref27] Graham M. B. Braciale V. L. Braciale T. J. (1994). Influenza virus-specific CD4+ T helper type 2 T lymphocytes do not promote recovery from experimental virus infection. J. Exp. Med. 180, 1273–1282. doi: 10.1084/jem.180.4.1273, PMID: 7931062 PMC2191682

[ref28] Guarda G. Braun M. Staehli F. Tardivel A. Mattmann C. Forster I.. (2011). Type I interferon inhibits interleukin-1 production and inflammasome activation. Immunity 34, 213–223. doi: 10.1016/j.immuni.2011.02.006, PMID: 21349431

[ref29] Guilliams M. De Kleer I. Henri S. Post S. Vanhoutte L. De Prijck S.. (2013). Alveolar macrophages develop from fetal monocytes that differentiate into long-lived cells in the first week of life via GM-CSF. J. Exp. Med. 210, 1977–1992. doi: 10.1084/jem.20131199, PMID: 24043763 PMC3782041

[ref30] Guilliams M. Svedberg F. R. (2021). Does tissue imprinting restrict macrophage plasticity? Nat. Immunol. 22, 118–127. doi: 10.1038/s41590-020-00849-2, PMID: 33462453

[ref31] Halder U. C. Bagchi P. Chattopadhyay S. Dutta D. Chawla-Sarkar M. (2011). Cell death regulation during influenza a virus infection by matrix (M1) protein: a model of viral control over the cellular survival pathway. Cell Death Dis. 2:e197. doi: 10.1038/cddis.2011.75, PMID: 21881599 PMC3186897

[ref32] Halstead E. S. Umstead T. M. Davies M. L. Kawasawa Y. I. Silveyra P. Howyrlak J.. (2018). GM-CSF overexpression after influenza a virus infection prevents mortality and moderates M1-like airway monocyte/macrophage polarization. Respir. Res. 19:3. doi: 10.1186/s12931-017-0708-5, PMID: 29304863 PMC5756339

[ref33] Hashimoto Y. Moki T. Takizawa T. Shiratsuchi A. Nakanishi Y. (2007). Evidence for phagocytosis of influenza virus-infected, apoptotic cells by neutrophils and macrophages in mice. J. Immunol. 178, 2448–2457. doi: 10.4049/jimmunol.178.4.2448, PMID: 17277152

[ref34] Hemann E. A. Green R. Turnbull J. B. Langlois R. A. Savan R. Gale M. Jr. (2019). Interferon-lambda modulates dendritic cells to facilitate T cell immunity during infection with influenza a virus. Nat. Immunol. 20, 1035–1045. doi: 10.1038/s41590-019-0408-z, PMID: 31235953 PMC6642690

[ref35] Hogner K. Wolff T. Pleschka S. Plog S. Gruber A. D. Kalinke U.. (2013). Macrophage-expressed IFN-beta contributes to apoptotic alveolar epithelial cell injury in severe influenza virus pneumonia. PLoS Pathog. 9:e1003188. doi: 10.1371/journal.ppat.1003188, PMID: 23468627 PMC3585175

[ref36] Hou F. Xiao K. Tang L. Xie L. (2021). Diversity of macrophages in lung homeostasis and diseases. Front. Immunol. 12:753940. doi: 10.3389/fimmu.2021.753940, PMID: 34630433 PMC8500393

[ref37] Huang S. Zhu B. Cheon I. S. Goplen N. P. Jiang L. Zhang R.. (2019). PPAR-gamma in macrophages limits pulmonary inflammation and promotes host recovery following respiratory viral infection. J. Virol. 93:e00030-19. doi: 10.1128/JVI.00030-19, PMID: 30787149 PMC6475778

[ref38] Hume P. S. Gibbings S. L. Jakubzick C. V. Tuder R. M. Curran-Everett D. Henson P. M.. (2020). Localization of macrophages in the human lung via design-based stereology. Am. J. Respir. Crit. Care Med. 201, 1209–1217. doi: 10.1164/rccm.201911-2105OC, PMID: 32197050 PMC7233346

[ref39] Hussell T. Bell T. J. (2014). Alveolar macrophages: plasticity in a tissue-specific context. Nat. Rev. Immunol. 14, 81–93. doi: 10.1038/nri3600, PMID: 24445666

[ref40] Ichinohe T. Pang I. K. Iwasaki A. (2010). Influenza virus activates inflammasomes via its intracellular M2 ion channel. Nat. Immunol. 11, 404–410. doi: 10.1038/ni.1861, PMID: 20383149 PMC2857582

[ref41] Imai Y. Kuba K. Neely G. G. Yaghubian-Malhami R. Perkmann T. van Loo G.. (2008). Identification of oxidative stress and toll-like receptor 4 signaling as a key pathway of acute lung injury. Cells 133, 235–249. doi: 10.1016/j.cell.2008.02.043, PMID: 18423196 PMC7112336

[ref42] Itano A. A. Jenkins M. K. (2003). Antigen presentation to naive CD4 T cells in the lymph node. Nat. Immunol. 4, 733–739. doi: 10.1038/ni957, PMID: 12888794

[ref43] Iwasaki A. (2016). Exploiting mucosal immunity for antiviral vaccines. Annu. Rev. Immunol. 34, 575–608. doi: 10.1146/annurev-immunol-032414-112315, PMID: 27168245

[ref44] Iwasaki A. Pillai P. S. (2014). Innate immunity to influenza virus infection. Nat. Rev. Immunol. 14, 315–328. doi: 10.1038/nri3665, PMID: 24762827 PMC4104278

[ref45] Iyer S. S. Ghaffari A. A. Cheng G. (2010). Lipopolysaccharide-mediated IL-10 transcriptional regulation requires sequential induction of type I IFNs and IL-27 in macrophages. J. Immunol. 185, 6599–6607. doi: 10.4049/jimmunol.1002041, PMID: 21041726 PMC4103176

[ref46] Jablonski K. A. Amici S. A. Webb L. M. Ruiz-Rosado Jde D. Popovich P. G. Partida-Sanchez S.. (2015). Novel markers to delineate murine M1 and M2 macrophages. PLoS One 10:e0145342. doi: 10.1371/journal.pone.0145342, PMID: 26699615 PMC4689374

[ref47] Jacobsen H. Walendy-Gnirss K. Tekin-Bubenheim N. Kouassi N. M. Ben-Batalla I. Berenbrok N.. (2021). Offspring born to influenza a virus infected pregnant mice have increased susceptibility to viral and bacterial infections in early life. Nat. Commun. 12:4957. doi: 10.1038/s41467-021-25220-3, PMID: 34400653 PMC8368105

[ref48] Janeway C. A. Jr. Medzhitov R. (2002). Innate immune recognition. Annu. Rev. Immunol. 20, 197–216. doi: 10.1146/annurev.immunol.20.083001.084359, PMID: 11861602

[ref49] Jayasekera J. P. Vinuesa C. G. Karupiah G. King N. J. C. (2006). Enhanced antiviral antibody secretion and attenuated immunopathology during influenza virus infection in nitric oxide synthase-2-deficient mice. J. Gen. Virol. 87, 3361–3371. doi: 10.1099/vir.0.82131-0, PMID: 17030871

[ref50] Jin B. Sun T. Yu X. H. Yang Y. X. Yeo A. E. (2012). The effects of TLR activation on T-cell development and differentiation. Clin. Dev. Immunol. 2012:836485. doi: 10.1155/2012/836485, PMID: 22737174 PMC3376488

[ref51] Kadl A. Bochkov V. N. Huber J. Leitinger N. (2004). Apoptotic cells as sources for biologically active oxidized phospholipids. Antioxid. Redox Signal. 6, 311–320. doi: 10.1089/152308604322899378, PMID: 15025932

[ref52] Karavitis J. Kovacs E. J. (2011). Macrophage phagocytosis: effects of environmental pollutants, alcohol, cigarette smoke, and other external factors. J. Leukoc. Biol. 90, 1065–1078. doi: 10.1189/jlb.0311114, PMID: 21878544 PMC3236556

[ref53] Karupiah G. Chen J. H. Nathan C. F. Mahalingam S. MacMicking J. D. (1998). Identification of nitric oxide synthase 2 as an innate resistance locus against ectromelia virus infection. J. Virol. 72, 7703–7706. doi: 10.1128/JVI.72.9.7703-7706.1998, PMID: 9696880 PMC110049

[ref54] Kim C. U. Jeong Y. J. Lee P. Lee M. S. Park J. H. Kim Y. S.. (2022). Extracellular nucleoprotein exacerbates influenza virus pathogenesis by activating toll-like receptor 4 and the NLRP3 inflammasome. Cell. Mol. Immunol. 19, 715–725. doi: 10.1038/s41423-022-00862-5, PMID: 35459853 PMC9026019

[ref55] Kim H. M. Lee Y. W. Lee K. J. Kim H. S. Cho S. W. van Rooijen N.. (2008). Alveolar macrophages are indispensable for controlling influenza viruses in lungs of pigs. J. Virol. 82, 4265–4274. doi: 10.1128/JVI.02602-07, PMID: 18287245 PMC2293066

[ref56] Klonoski J. M. Watson T. Bickett T. E. Svendsen J. M. Gau T. J. Britt A.. (2018). Contributions of influenza virus hemagglutinin and host immune responses toward the severity of influenza virus: *Streptococcus pyogenes* superinfections. Viral Immunol. 31, 457–469. doi: 10.1089/vim.2017.0193, PMID: 29870311 PMC6043403

[ref57] Koutsakos M. McWilliam H. E. G. Aktepe T. E. Fritzlar S. Illing P. T. Mifsud N. A.. (2019). Downregulation of MHC class I expression by influenza a and B viruses. Front. Immunol. 10:1158. doi: 10.3389/fimmu.2019.01158, PMID: 31191533 PMC6548845

[ref58] Kudva A. Scheller E. V. Robinson K. M. Crowe C. R. Choi S. M. Slight S. R.. (2011). Influenza a inhibits Th17-mediated host defense against bacterial pneumonia in mice. J. Immunol. 186, 1666–1674. doi: 10.4049/jimmunol.1002194, PMID: 21178015 PMC4275066

[ref59] Kulikauskaite J. Wack A. (2020). Teaching old dogs new tricks? The plasticity of lung alveolar macrophage subsets. Trends Immunol. 41, 864–877. doi: 10.1016/j.it.2020.08.008, PMID: 32896485 PMC7472979

[ref60] Kuriakose T. Man S. M. Malireddi R. K. Karki R. Kesavardhana S. Place D. E.. (2016). ZBP1/DAI is an innate sensor of influenza virus triggering the NLRP3 inflammasome and programmed cell death pathways. Sci. Immunol. 1:aag2045. doi: 10.1126/sciimmunol.aag2045, PMID: 27917412 PMC5131924

[ref61] Landsman L. Jung S. (2007). Lung macrophages serve as obligatory intermediate between blood monocytes and alveolar macrophages. J. Immunol. 179, 3488–3494. doi: 10.4049/jimmunol.179.6.3488, PMID: 17785782

[ref62] Lauzon-Joset J. F. Scott N. M. Mincham K. T. Stumbles P. A. Holt P. G. Strickland D. H. (2019). Pregnancy induces a steady-state shift in alveolar macrophage M1/M2 phenotype that is associated with a heightened severity of influenza virus infection: mechanistic insight using mouse models. J. Infect. Dis. 219, 1823–1831. doi: 10.1093/infdis/jiy732, PMID: 30576502

[ref63] Lee S. M. Gardy J. L. Cheung C. Y. Cheung T. K. Hui K. P. Ip N. Y.. (2009). Systems-level comparison of host-responses elicited by avian H5N1 and seasonal H1N1 influenza viruses in primary human macrophages. PLoS One 4:e8072. doi: 10.1371/journal.pone.0008072, PMID: 20011590 PMC2788213

[ref64] Liegeois M. Legrand C. Desmet C. J. Marichal T. Bureau F. (2018). The interstitial macrophage: a long-neglected piece in the puzzle of lung immunity. Cell. Immunol. 330, 91–96. doi: 10.1016/j.cellimm.2018.02.001, PMID: 29458975

[ref65] Lin K. L. Suzuki Y. Nakano H. Ramsburg E. Gunn M. D. (2008). CCR2+ monocyte-derived dendritic cells and exudate macrophages produce influenza-induced pulmonary immune pathology and mortality. J. Immunol. 180, 2562–2572. doi: 10.4049/jimmunol.180.4.2562, PMID: 18250467

[ref66] Liu G. Lu Y. Thulasi Raman S. N. Xu F. Wu Q. Li Z.. (2018). Nuclear-resident RIG-I senses viral replication inducing antiviral immunity. Nat. Commun. 9:3199. doi: 10.1038/s41467-018-05745-w, PMID: 30097581 PMC6086882

[ref67] Liu R. Sheng Z. Huang C. Wang D. Li F. (2020). Influenza D virus. Curr. Opin. Virol. 44, 154–161. doi: 10.1016/j.coviro.2020.08.004, PMID: 32932215 PMC7755673

[ref68] Liu Q. Zhou Y. H. Yang Z. Q. (2016). The cytokine storm of severe influenza and development of immunomodulatory therapy. Cell. Mol. Immunol. 13, 3–10. doi: 10.1038/cmi.2015.74, PMID: 26189369 PMC4711683

[ref69] Londrigan S. L. Short K. R. Ma J. Gillespie L. Rockman S. P. Brooks A. G.. (2015). Infection of mouse macrophages by seasonal influenza viruses can be restricted at the level of virus entry and at a late stage in the virus life cycle. J. Virol. 89, 12319–12329. doi: 10.1128/JVI.01455-15, PMID: 26423941 PMC4665238

[ref70] Lund J. M. Alexopoulou L. Sato A. Karow M. Adams N. C. Gale N. W.. (2004). Recognition of single-stranded RNA viruses by toll-like receptor 7. Proc. Natl. Acad. Sci. U. S. A. 101, 5598–5603. doi: 10.1073/pnas.0400937101, PMID: 15034168 PMC397437

[ref71] Lv J. Wang Z. Qu Y. Zhu H. Zhu Q. Tong W.. (2021). Distinct uptake, amplification, and release of SARS-CoV-2 by M1 and M2 alveolar macrophages. Cell Discov. 7:24. doi: 10.1038/s41421-021-00258-1, PMID: 33850112 PMC8043100

[ref72] MacLean A. J. Richmond N. Koneva L. Attar M. Medina C. A. P. Thornton E. E.. (2022). Secondary influenza challenge triggers resident memory B cell migration and rapid relocation to boost antibody secretion at infected sites. Immunity 55, 718–733.e8. doi: 10.1016/j.immuni.2022.03.003, PMID: 35349789 PMC9044924

[ref73] Maines T. R. Szretter K. J. Perrone L. Belser J. A. Bright R. A. Zeng H.. (2008). Pathogenesis of emerging avian influenza viruses in mammals and the host innate immune response. Immunol. Rev. 225, 68–84. doi: 10.1111/j.1600-065X.2008.00690.x, PMID: 18837776

[ref74] Makris S. Paulsen M. Johansson C. (2017). Type I interferons as regulators of lung inflammation. Front. Immunol. 8:259. doi: 10.3389/fimmu.2017.00259, PMID: 28344581 PMC5344902

[ref75] Mallampalli R. K. Adair J. Elhance A. Farkas D. Chafin L. Long M. E.. (2021). Interferon lambda Signaling in macrophages is necessary for the antiviral response to influenza. Front. Immunol. 12:735576. doi: 10.3389/fimmu.2021.735576, PMID: 34899695 PMC8655102

[ref76] Mantovani A. Sica A. Sozzani S. Allavena P. Vecchi A. Locati M. (2004). The chemokine system in diverse forms of macrophage activation and polarization. Trends Immunol. 25, 677–686. doi: 10.1016/j.it.2004.09.015, PMID: 15530839

[ref77] Maroof A. Yorgensen Y. M. Li Y. Evans J. T. (2014). Intranasal vaccination promotes detrimental Th17-mediated immunity against influenza infection. PLoS Pathog. 10:e1003875. doi: 10.1371/journal.ppat.1003875, PMID: 24465206 PMC3900655

[ref78] Martinez-Colon G. J. Warheit-Niemi H. Gurczynski S. J. Taylor Q. M. Wilke C. A. Podsiad A. B.. (2019). Influenza-induced immune suppression to methicillin-resistant *Staphylococcus aureus* is mediated by TLR9. PLoS Pathog. 15:e1007560. doi: 10.1371/journal.ppat.1007560, PMID: 30682165 PMC6364947

[ref79] Marvin S. A. Russier M. Huerta C. T. Russell C. J. Schultz-Cherry S. (2017). Influenza virus overcomes cellular blocks to productively replicate. Impact. Macrophage Funct. J. Virol. 91:e01417-16. doi: 10.1128/JVI.01417-16, PMID: 27807237 PMC5215328

[ref80] McKinstry K. K. Strutt T. M. Buck A. Curtis J. D. Dibble J. P. Huston G.. (2009). IL-10 deficiency unleashes an influenza-specific Th17 response and enhances survival against high-dose challenge. J. Immunol. 182, 7353–7363. doi: 10.4049/jimmunol.0900657, PMID: 19494257 PMC2724021

[ref81] Meesmann H. M. Fehr E. M. Kierschke S. Herrmann M. Bilyy R. Heyder P.. (2010). Decrease of sialic acid residues as an eat-me signal on the surface of apoptotic lymphocytes. J. Cell Sci. 123, 3347–3356. doi: 10.1242/jcs.066696, PMID: 20826457

[ref82] Misharin A. V. Morales-Nebreda L. Mutlu G. M. Budinger G. R. Perlman H. (2013). Flow cytometric analysis of macrophages and dendritic cell subsets in the mouse lung. Am. J. Respir. Cell Mol. Biol. 49, 503–510. doi: 10.1165/rcmb.2013-0086MA, PMID: 23672262 PMC3824047

[ref83] Miyauchi K. (2017). Helper T cell responses to respiratory viruses in the lung: development, virus suppression, and pathogenesis. Viral Immunol. 30, 421–430. doi: 10.1089/vim.2017.0018, PMID: 28650258

[ref84] Mordstein M. Neugebauer E. Ditt V. Jessen B. Rieger T. Falcone V.. (2010). Lambda interferon renders epithelial cells of the respiratory and gastrointestinal tracts resistant to viral infections. J. Virol. 84, 5670–5677. doi: 10.1128/JVI.00272-10, PMID: 20335250 PMC2876583

[ref85] Morens D. M. Taubenberger J. K. Fauci A. S. (2008). Predominant role of bacterial pneumonia as a cause of death in pandemic influenza: implications for pandemic influenza preparedness. J. Infect. Dis. 198, 962–970. doi: 10.1086/591708, PMID: 18710327 PMC2599911

[ref86] Neupane A. S. Willson M. Chojnacki A. K. Vargas E. S. C. F. Morehouse C. Carestia A.. (2020). Patrolling alveolar macrophages conceal bacteria from the immune system to maintain homeostasis. Cells 183, 110–125.e11. doi: 10.1016/j.cell.2020.08.020, PMID: 32888431

[ref87] Nita-Lazar M. Banerjee A. Feng C. Amin M. N. Frieman M. B. Chen W. H.. (2015). Desialylation of airway epithelial cells during influenza virus infection enhances pneumococcal adhesion via galectin binding. Mol. Immunol. 65, 1–16. doi: 10.1016/j.molimm.2014.12.010, PMID: 25597246 PMC4344939

[ref88] Oshansky C. M. Pickens J. A. Bradley K. C. Jones L. P. Saavedra-Ebner G. M. Barber J. P.. (2011). Avian influenza viruses infect primary human bronchial epithelial cells unconstrained by sialic acid alpha2,3 residues. PLoS One 6:e21183. doi: 10.1371/journal.pone.0021183, PMID: 21731666 PMC3121740

[ref89] Rapoport E. M. Mochalova L. V. Gabius H. J. Romanova J. Bovin N. V. (2006). Search for additional influenza virus to cell interactions. Glycoconj. J. 23, 115–125. doi: 10.1007/s10719-006-5444-x, PMID: 16575529

[ref90] Robinson K. M. Lee B. Scheller E. V. Mandalapu S. Enelow R. I. Kolls J. K.. (2015). The role of IL-27 in susceptibility to post-influenza *Staphylococcus aureus* pneumonia. Respir. Res. 16:10. doi: 10.1186/s12931-015-0168-8, PMID: 25651926 PMC4324414

[ref91] Roche P. A. Furuta K. (2015). The ins and outs of MHC class II-mediated antigen processing and presentation. Nat. Rev. Immunol. 15, 203–216. doi: 10.1038/nri3818, PMID: 25720354 PMC6314495

[ref92] Rodgers B. C. Mims C. A. (1982). Influenza virus replication in human alveolar macrophages. J. Med. Virol. 9, 177–184. doi: 10.1002/jmv.1890090304, PMID: 7097255

[ref93] Roszer T. (2015). Understanding the mysterious M2 macrophage through activation markers and effector mechanisms. Mediat. Inflamm. 2015:816460. doi: 10.1155/2015/816460, PMID: 26089604 PMC4452191

[ref94] Sato T. Onai N. Yoshihara H. Arai F. Suda T. Ohteki T. (2009). Interferon regulatory factor-2 protects quiescent hematopoietic stem cells from type I interferon-dependent exhaustion. Nat. Med. 15, 696–700. doi: 10.1038/nm.1973, PMID: 19483695

[ref95] Schneider C. Nobs S. P. Heer A. K. Kurrer M. Klinke G. van Rooijen N.. (2014a). Alveolar macrophages are essential for protection from respiratory failure and associated morbidity following influenza virus infection. PLoS Pathog. 10:e1004053. doi: 10.1371/journal.ppat.1004053, PMID: 24699679 PMC3974877

[ref96] Schneider C. Nobs S. P. Kurrer M. Rehrauer H. Thiele C. Kopf M. (2014b). Induction of the nuclear receptor PPAR-gamma by the cytokine GM-CSF is critical for the differentiation of fetal monocytes into alveolar macrophages. Nat. Immunol. 15, 1026–1037. doi: 10.1038/ni.3005, PMID: 25263125

[ref97] Schulz O. Diebold S. S. Chen M. Naslund T. I. Nolte M. A. Alexopoulou L.. (2005). Toll-like receptor 3 promotes cross-priming to virus-infected cells. Nature 433, 887–892. doi: 10.1038/nature03326, PMID: 15711573

[ref98] Serbina N. V. Pamer E. G. (2006). Monocyte emigration from bone marrow during bacterial infection requires signals mediated by chemokine receptor CCR2. Nat. Immunol. 7, 311–317. doi: 10.1038/ni1309, PMID: 16462739

[ref99] Shahangian A. Chow E. K. Tian X. Kang J. R. Ghaffari A. Liu S. Y.. (2009). Type I IFNs mediate development of postinfluenza bacterial pneumonia in mice. J. Clin. Invest. 119, 1910–1920. doi: 10.1172/JCI35412, PMID: 19487810 PMC2701856

[ref100] Shepardson K. Larson K. Cho H. Johns L. L. Malkoc Z. Stanek K.. (2019). A novel role for PDZ-binding motif of influenza a virus nonstructural protein 1 in regulation of host susceptibility to Postinfluenza bacterial superinfections. Viral Immunol. 32, 131–143. doi: 10.1089/vim.2018.0118, PMID: 30822217 PMC6479245

[ref101] Shiratsuchi A. Kaido M. Takizawa T. Nakanishi Y. (2000). Phosphatidylserine-mediated phagocytosis of influenza a virus-infected cells by mouse peritoneal macrophages. J. Virol. 74, 9240–9244. doi: 10.1128/JVI.74.19.9240-9244.2000, PMID: 10982371 PMC102123

[ref102] Shiratsuchi A. Nakanishi Y. (2006). Elimination of influenza virus-infected cells by phagocytosis. Yakugaku Zasshi 126, 1245–1251. doi: 10.1248/yakushi.126.1245, PMID: 17139150

[ref103] Shirey K. A. Lai W. Patel M. C. Pletneva L. M. Pang C. Kurt-Jones E.. (2016). Novel strategies for targeting innate immune responses to influenza. Mucosal Immunol. 9, 1173–1182. doi: 10.1038/mi.2015.141, PMID: 26813341 PMC5125448

[ref104] Siegel S. J. Roche A. M. Weiser J. N. (2014). Influenza promotes pneumococcal growth during coinfection by providing host sialylated substrates as a nutrient source. Cell Host Microbe 16, 55–67. doi: 10.1016/j.chom.2014.06.005, PMID: 25011108 PMC4096718

[ref105] Skehel J. J. Wiley D. C. (2000). Receptor binding and membrane fusion in virus entry: the influenza hemagglutinin. Annu. Rev. Biochem. 69, 531–569. doi: 10.1146/annurev.biochem.69.1.531, PMID: 10966468

[ref106] Smith A. M. McCullers J. A. (2014). Secondary bacterial infections in influenza virus infection pathogenesis. Curr. Top. Microbiol. Immunol. 385, 327–356. doi: 10.1007/82_2014_394, PMID: 25027822 PMC7122299

[ref107] Stein M. Keshav S. Harris N. Gordon S. (1992). Interleukin 4 potently enhances murine macrophage mannose receptor activity: a marker of alternative immunologic macrophage activation. J. Exp. Med. 176, 287–292. doi: 10.1084/jem.176.1.287, PMID: 1613462 PMC2119288

[ref108] Sun K. Metzger D. W. (2008). Inhibition of pulmonary antibacterial defense by interferon-gamma during recovery from influenza infection. Nat. Med. 14, 558–564. doi: 10.1038/nm1765, PMID: 18438414

[ref109] Taubenberger J. K. Morens D. M. (2008). The pathology of influenza virus infections. Annu. Rev. Pathol. 3, 499–522. doi: 10.1146/annurev.pathmechdis.3.121806.154316, PMID: 18039138 PMC2504709

[ref110] Teijaro J. R. Ng C. Lee A. M. Sullivan B. M. Sheehan K. C. Welch M.. (2013). Persistent LCMV infection is controlled by blockade of type I interferon signaling. Science 340, 207–211. doi: 10.1126/science.1235214, PMID: 23580529 PMC3640797

[ref111] Thompson C. I. Barclay W. S. Zambon M. C. Pickles R. J. (2006). Infection of human airway epithelium by human and avian strains of influenza a virus. J. Virol. 80, 8060–8068. doi: 10.1128/JVI.00384-06, PMID: 16873262 PMC1563802

[ref112] Tisoncik J. R. Korth M. J. Simmons C. P. Farrar J. Martin T. R. Katze M. G. (2012). Into the eye of the cytokine storm. Microbiol. Mol. Biol. Rev. 76, 16–32. doi: 10.1128/MMBR.05015-11, PMID: 22390970 PMC3294426

[ref113] Tong S. Zhu X. Li Y. Shi M. Zhang J. Bourgeois M.. (2013). New world bats harbor diverse influenza a viruses. PLoS Pathog. 9:e1003657. doi: 10.1371/journal.ppat.1003657, PMID: 24130481 PMC3794996

[ref114] Tripathi S. Batra J. Cao W. Sharma K. Patel J. R. Ranjan P.. (2013). Influenza a virus nucleoprotein induces apoptosis in human airway epithelial cells: implications of a novel interaction between nucleoprotein and host protein Clusterin. Cell Death Dis. 4:e562. doi: 10.1038/cddis.2013.89, PMID: 23538443 PMC3615740

[ref115] Tumpey T. M. Garcia-Sastre A. Taubenberger J. K. Palese P. Swayne D. E. Pantin-Jackwood M. J.. (2005). Pathogenicity of influenza viruses with genes from the 1918 pandemic virus: functional roles of alveolar macrophages and neutrophils in limiting virus replication and mortality in mice. J. Virol. 79, 14933–14944. doi: 10.1128/JVI.79.23.14933-14944.2005, PMID: 16282492 PMC1287592

[ref116] Ural B. B. Yeung S. T. Damani-Yokota P. Devlin J. C. de Vries M. Vera-Licona P.. (2020). Identification of a nerve-associated, lung-resident interstitial macrophage subset with distinct localization and immunoregulatory properties. Sci. Immunol. 5:eaax8756. doi: 10.1126/sciimmunol.aax8756, PMID: 32220976 PMC7717505

[ref117] van Riel D. Leijten L. M. van der Eerden M. Hoogsteden H. C. Boven L. A. Lambrecht B. N.. (2011). Highly pathogenic avian influenza virus H5N1 infects alveolar macrophages without virus production or excessive TNF-alpha induction. PLoS Pathog. 7:e1002099. doi: 10.1371/journal.ppat.1002099, PMID: 21731493 PMC3121882

[ref118] Waffarn E. E. Baumgarth N. (2011). Protective B cell responses to flu--no fluke! J. Immunol. 186, 3823–3829. doi: 10.4049/jimmunol.1002090, PMID: 21422252 PMC3154207

[ref119] Wang Y. Li T. Chen Y. Wei H. Sun R. Tian Z. (2017). Involvement of NK cells in IL-28B-mediated immunity against influenza virus infection. J. Immunol. 199, 1012–1020. doi: 10.4049/jimmunol.1601430, PMID: 28637903

[ref120] Wang Z. Li S. Huang B. (2022). Alveolar macrophages: Achilles' heel of SARS-CoV-2 infection. Signal Transduct. Target. Ther. 7:242. doi: 10.1038/s41392-022-01106-8, PMID: 35853858 PMC9295089

[ref121] Wang J. Nikrad M. P. Travanty E. A. Zhou B. Phang T. Gao B.. (2012). Innate immune response of human alveolar macrophages during influenza a infection. PLoS One 7:e29879. doi: 10.1371/journal.pone.0053383, PMID: 22396727 PMC3292548

[ref122] Wang J. Oberley-Deegan R. Wang S. Nikrad M. Funk C. J. Hartshorn K. L.. (2009). Differentiated human alveolar type II cells secrete antiviral IL-29 (IFN-lambda 1) in response to influenza a infection. J. Immunol. 182, 1296–1304. doi: 10.4049/jimmunol.182.3.1296, PMID: 19155475 PMC4041086

[ref123] Wang X. Yin X. Zhang B. Liu C. Lin Y. Huang X.. (2022). A prophylactic effect of aluminium-based adjuvants against respiratory viruses via priming local innate immunity. Emerg. Microbes Infect. 11, 914–925. doi: 10.1080/22221751.2022.2050951, PMID: 35254215 PMC8967214

[ref124] Watanabe Y. Hashimoto Y. Shiratsuchi A. Takizawa T. Nakanishi Y. (2005). Augmentation of fatality of influenza in mice by inhibition of phagocytosis. Biochem. Biophys. Res. Commun. 337, 881–886. doi: 10.1016/j.bbrc.2005.09.133, PMID: 16216222

[ref125] Watanabe Y. Shiratsuchi A. Shimizu K. Takizawa T. Nakanishi Y. (2002). Role of phosphatidylserine exposure and sugar chain desialylation at the surface of influenza virus-infected cells in efficient phagocytosis by macrophages. J. Biol. Chem. 277, 18222–18228. doi: 10.1074/jbc.M201074200, PMID: 11884410

[ref126] Wei F. Gao C. Wang Y. (2022). The role of influenza a virus-induced hypercytokinemia. Crit. Rev. Microbiol. 48, 240–256. doi: 10.1080/1040841X.2021.1960482, PMID: 34353210

[ref127] Wiley D. C. Skehel J. J. (1987). The structure and function of the hemagglutinin membrane glycoprotein of influenza virus. Annu. Rev. Biochem. 56, 365–394. doi: 10.1146/annurev.bi.56.070187.002053, PMID: 3304138

[ref128] Wong C. K. Smith C. A. Sakamoto K. Kaminski N. Koff J. L. Goldstein D. R. (2017). Aging impairs alveolar macrophage phagocytosis and increases influenza-induced mortality in mice. J. Immunol. 199, 1060–1068. doi: 10.4049/jimmunol.1700397, PMID: 28646038 PMC5557035

[ref129] Xia C. Xu W. Ai X. Zhu Y. Geng P. Niu Y.. (2022). Autophagy and exosome Coordinately enhance macrophage M1 polarization and recruitment in influenza a virus infection. Front. Immunol. 13:722053. doi: 10.3389/fimmu.2022.926781, PMID: 35371077 PMC8967985

[ref130] Xing Z. Afkhami S. Bavananthasivam J. Fritz D. K. D'Agostino M. R. Vaseghi-Shanjani M.. (2020). Innate immune memory of tissue-resident macrophages and trained innate immunity: re-vamping vaccine concept and strategies. J. Leukoc. Biol. 108, 825–834. doi: 10.1002/JLB.4MR0220-446R, PMID: 32125045

[ref131] Yao D. Bao L. Li F. Liu B. Wu X. Hu Z.. (2022). H1N1 influenza virus dose dependent induction of dysregulated innate immune responses and STAT1/3 activation are associated with pulmonary immunopathological damage. Virulence 13, 1558–1572. doi: 10.1080/21505594.2022.2120951, PMID: 36082929 PMC9467583

[ref132] Yona S. Kim K. W. Wolf Y. Mildner A. Varol D. Breker M.. (2013). Fate mapping reveals origins and dynamics of monocytes and tissue macrophages under homeostasis. Immunity 38, 79–91. doi: 10.1016/j.immuni.2012.12.001, PMID: 23273845 PMC3908543

[ref133] Yu X. Buttgereit A. Lelios I. Utz S. G. Cansever D. Becher B.. (2017). The cytokine TGF-beta promotes the development and homeostasis of alveolar macrophages. Immunity 47, 903–912 e4. doi: 10.1016/j.immuni.2017.10.007, PMID: 29126797

[ref134] Yu W. C. Chan R. W. Wang J. Travanty E. A. Nicholls J. M. Peiris J. S.. (2011). Viral replication and innate host responses in primary human alveolar epithelial cells and alveolar macrophages infected with influenza H5N1 and H1N1 viruses. J. Virol. 85, 6844–6855. doi: 10.1128/JVI.02200-10, PMID: 21543489 PMC3126566

[ref135] Zaynagetdinov R. Sherrill T. P. Kendall P. L. Segal B. H. Weller K. P. Tighe R. M.. (2013). Identification of myeloid cell subsets in murine lungs using flow cytometry. Am. J. Respir. Cell Mol. Biol. 49, 180–189. doi: 10.1165/rcmb.2012-0366MA, PMID: 23492192 PMC3824033

[ref136] Zhang N. Bao Y. J. Tong A. H. Zuyderduyn S. Bader G. D. Malik Peiris J. S.. (2018). Whole transcriptome analysis reveals differential gene expression profile reflecting macrophage polarization in response to influenza a H5N1 virus infection. BMC Med. Genet. 11:20. doi: 10.1186/s12920-018-0335-0, PMID: 29475453 PMC6389164

[ref137] Zhao X. Dai J. Xiao X. Wu L. Zeng J. Sheng J.. (2014). PI3K/Akt signaling pathway modulates influenza virus induced mouse alveolar macrophage polarization to M1/M2b. PLoS One 9:e104506. doi: 10.1371/journal.pone.0115872, PMID: 25105760 PMC4126709

[ref138] Zhao Y. Zou W. Du J. Zhao Y. (2018). The origins and homeostasis of monocytes and tissue-resident macrophages in physiological situation. J. Cell. Physiol. 233, 6425–6439. doi: 10.1002/jcp.26461, PMID: 29323706

[ref139] Zhou X. Moore B. B. (2018). Location or origin? What is critical for macrophage propagation of lung fibrosis? Eur. Respir. J. 51:1800103. doi: 10.1183/13993003.00103-2018, PMID: 29496789 PMC6383715

[ref140] Zhu B. Wu Y. Huang S. Zhang R. Son Y. M. Li C.. (2021). Uncoupling of macrophage inflammation from self-renewal modulates host recovery from respiratory viral infection. Immunity 54, 1200–1218.e9. doi: 10.1016/j.immuni.2021.04.001, PMID: 33951416 PMC8192557

[ref141] Zhu J. Yamane H. Paul W. E. (2010). Differentiation of effector CD4 T cell populations (*). Annu. Rev. Immunol. 28, 445–489. doi: 10.1146/annurev-immunol-030409-101212, PMID: 20192806 PMC3502616

